# General practice variation when initiating long-term prescribing of proton pump inhibitors: a nationwide cohort study

**DOI:** 10.1186/s12875-016-0460-9

**Published:** 2016-05-28

**Authors:** P. F. Haastrup, S. Rasmussen, J. M. Hansen, R. D. Christensen, J. Søndergaard, D. E. Jarbøl

**Affiliations:** Research Unit of General Practice, Department of Public Health, University of Southern Denmark, Odense, Denmark; Department of Medical Gastroenterology, Odense University Hospital, Odense, Denmark

**Keywords:** General practice variation, Proton pump inhibitors, Prescribing patterns

## Abstract

**Background:**

Suggestions of overprescribing of proton pump inhibitors (PPIs) for long-term treatment in primary care have been raised. This study aims to analyse associations between general practice characteristics and initiating long-term treatment with PPIs.

**Methods:**

A nationwide register-based cohort study of patients over 18 years redeeming first-time prescription for PPI issued by a general practitioner in Denmark in 2011. Patients redeeming more than 60 defined daily doses (DDDs) of PPI within six months were defined first-time long-term users. Detailed information on diagnoses, concomitant drug use and sociodemography of the cohort was extracted. Practice characteristics such as age and gender of the general practitioner (GP), number of GPs, number of patients per GP, geographical location and training practice status were linked to each PPI user. Logistic regression analysis was used to determine associations between practice characteristics and initiating long-term prescribing of PPIs.

**Results:**

We identified 90 556 first-time users of PPI. A total of 30 963 (34.2 %) met criteria for long-term use at six months follow-up. GPs over 65 years had significantly higher odds of long-term prescribing (OR 1.32, CI 1.16-1.50), when compared to younger GPs (<45 years). Furthermore, female GPs were significantly less likely to prescribe long-term treatment with PPIs (OR 0.87, CI 0.81-0.93) compared to male GPs.

**Conclusions:**

Practice characteristics such as GP age and gender could explain some of the observed variation in prescribing patterns for PPIs. This variation may indicate a potential for enhancing rational prescribing of PPIs.

## Background

Prescribing of proton pump inhibitors (PPIs) has increased significantly over the past decades and the vast majority is redeemed in primary care [1]. An increasing number of patients appear to be treated with PPIs for longer periods of time [1], and it has been stated that PPIs are prescribed and continued for questionable reasons [2–4]. Guidelines to promote rational management of patients with dyspepsia and rational prescribing of PPIs have been introduced [5, 6], but do not seem to have influenced the increasing prescribing substantially [1].

Patients are prescribed increasing quantities of PPI on an empirical background when initiating treatment [7]. A few weeks of treatment can cause acid rebound hypersecretion and acid related symptoms after withdrawal [8, 9]. Therefore, prescribing of PPIs for ambiguous reasons for more than a few weeks entails the risk of creating a vicious circle increasing the need for PPIs. Hence, moderation when initiating PPI treatment for unclear reasons is warranted.

Several factors might influence the prescribing pattern of PPIs. It has been demonstrated that patient-related factors have some impact on the prescribing of PPIs [7, 10, 11], but physician-related factors might be of importance as well. Studies have shown that general practice characteristics such as practice size, organisation in partnership or single-handed practices, the degree of urbanisation and having training practice status influence management of patients [12–17]. How general practice factors are associated with initiating long-term treatment with PPIs have not been analysed. Identifying practice characteristics of importance for initiating long-term PPI therapy may have implications for future organisation of primary care services and can help develop targeted interventions to enhance rational prescribing. Therefore, the aim of this study was to analyse associations between practice characteristics and initiation of long-term treatment with PPIs.

## Methods

This study is a register-based cohort study of the entire Danish population of approximately 5.6 million people and all general practices in Denmark (approximately 2200 practices [18]). The Danish health care system is tax-funded, and more than 98 % of the inhabitants are registered with a GP, who acts as gatekeeper, performing initial diagnostics and treatment and referring patients to secondary care when required. All citizens have free and equal access to health care services [18].

All inhabitants are assigned a unique civil registration number, and each general practice is registered with their own identification number. These identification numbers enable accurate linkage of patients and general practices through all national registers [19].

### Patient cohort sampling

**Fig. 1:**
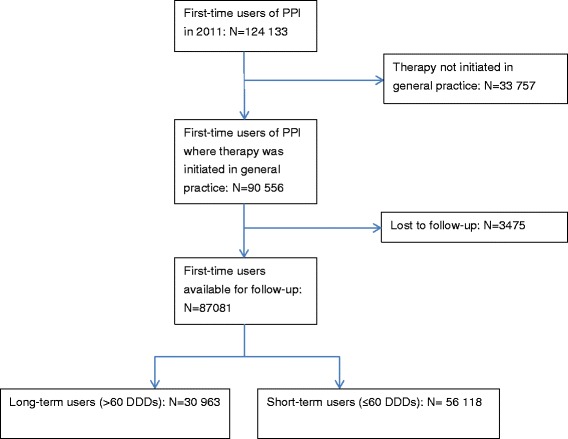
Flow-chart of patient cohort sampling

In the Danish National Prescription Register [20] we identified all individuals over 18 years redeeming a prescription for PPI in 2011. The date of the first redemption of PPI in 2011 was defined the index date. In order to include only incident PPI users, patients having redeemed PPIs within six years prior to the index date were excluded. The cohort was followed for six months, and similar to other studies [21, 22], patients redeeming >60 DDDs within six months were defined incident long-term users (Fig. 1). Patient factors in terms of gastrointestinal morbidity [23], use of ulcerogenic medication [23], general comorbidity [10] and socioeconomic status [10, 11] have in previous studies been associated with use of PPIs. Therefore, we retrieved additional data on diagnoses from the Danish National Patient Register [24], the Danish National Prescription Register and data on income, cohabitation status and highest attained education from sociodemographic registers contained in Statistics Denmark [25, 26]. Drugs were defined as comedication, if the patient had redeemed a quantity covering the date of redeeming the PPI. Morbidity was analysed as gastrointestinal morbidity (diagnoses of peptic ulcer, gastrooesophageal reflux disease (including oesophagitis), functional dyspepsia) and as general comorbidity measured by the Charlson Comorbidity Index [27].

### General practice data

**Fig. 2:**
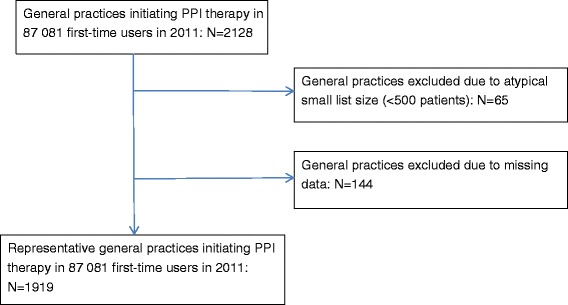
Flow-chart of sampling of general practices initiating PPI therapy in first-time users in 2011

The prescriptions for PPI were linked to the prescriber through the Danish National Health Service Provider Register. We extracted data on the general practices prescribing PPIs to the cohort of first-time users. The average list size of a typical Danish GP is 1600 patients [18], and practices with atypical small list sizes (<500 patients) were excluded, because they were thought not to be representative (Fig. 2). Practices with missing data in 2010 or 2011 were omitted as well, as this indicates that these practices were established or closed in that period (Fig. 2).

We identified the number of doctors registered at each practice and catagorised them as established GPs or temporary doctors. Doctors not registered in the entire period were defined temporary doctors. Practices were defined single-handed practices, if only one established GP was registered, and partnership practice if two or more established GPs were registered. The majority of temporary doctors in general practice are junior doctors having a six- to twelve-month residency, and practices with these doctors listed were defined training practices. We calculated the number of patients per GP by dividing the practice’s patient list size by the number of established GPs within the practice. In single-handed practices the age and gender of the GP were retrieved, and in partnership practices we calculated the mean age of the established group of GPs and assessed, whether the group of established GPs comprised exclusively men or women, predominantly men or women or equally mixed.

The postcode of all practices was extracted. Practices within the greater Copenhagen postcode area were categorised as capital area practices. Practices outside the capital area, but in a postcode comprising a city with more than 10 000 inhabitants, were labelled provincial city practices. All other practice locations were categorised as rural [28]. According to Danish legislation neither the individual GP nor the individual patient should be identifiable for the researchers. Therefore, a few practices (*N* = 4) with postcode comprising less than three other practices had missing postcode and could not be characterised according to degree of urbanisation.

For each practice we calculated a *“long-term user proportion”* defined as the proportion of incident users of PPIs within the practice who fulfilled the criteria for long-term use six months after the initial prescription (>60 DDD).

### Statistics

The analyses were conducted both with the entire cohort of general practices and with stratification into single-handed and partnership practices. This was done because the variables age and gender were exact values in single-handed practices, but average values in partnership practices. Mixed effects logistic regression models with patients nested within practice were used to calculate odds ratios (ORs) with 95 % confidence intervals (CI) for associations between long-term prescribing of PPIs and practice characteristics. Two regression models were used. Model one estimated the crude OR for the association of each practice characteristic and prescribing of long-term treatment with PPIs. Model two estimated the OR for each practice characteristic, adjusted for both patient characteristics and other practice characteristics included in the analyses. Patient characteristics adjusted for were age, gender, gastrointestinal morbidity, socioeconomic status (income, highest attained education and cohabitation status), comedication with non-steroidal anti-inflammatory drugs (NSAIDs), antiplatelet drugs, anticoagulants, selective serotonine reuptake inhibitors (SSRIs) and comorbidity [7].

A sensitivity analysis with a definition of long-term use increased to 90 DDDs within six months was performed in order to explore the consistency of the associations when changing threshold for long-term use.

*P*-values <0.05 were considered statistically significant associations. All analyses were performed using STATA12 (STATACorp, College Station, TX, USA).

### Ethics

According to “The Act on Research Ethics Review of Health Research Projects in Denmark” this register-based study did not require approval by the Research Ethics Committee. The study was approved by the Danish Data Protection Agency, J. nr. 2012-41-0280. Neither patients nor GPs were identifiable in the dataset.

## Results

We identified 124 133 adult first-time users of PPI in 2011. For 90 556 (72.9 %) of the first-time users the prescription for PPI was issued in general practice. Six months after initial redemption of PPIs the cohort was subdivided into short-term and long-term users (Fig. 1). A total of 3475 individuals were lost to follow-up, because they had either died or moved abroad within the six months. Hence, their use of PPI in the observation period could not be assessed and they were excluded. A total of 30 963 (35.5 %) of the 87 081 first-time users starting therapy initiated in general practice and available for follow-up met criteria for long-term use (the *“long-term user proportion”*) (Fig. 1).

The prescriptions for PPI to the cohort of first-time users derived from 2128 general practices. A total of 65 practices were excluded due to atypical small list size (<500 patients). A total of 144 practices were excluded due to missing data, indicating that they might have been established or closed down in 2010 or 2011 (Fig. 2). Characteristics of the 1919 representative practices initiating PPI therapy were obtained.

**Fig. 3:**
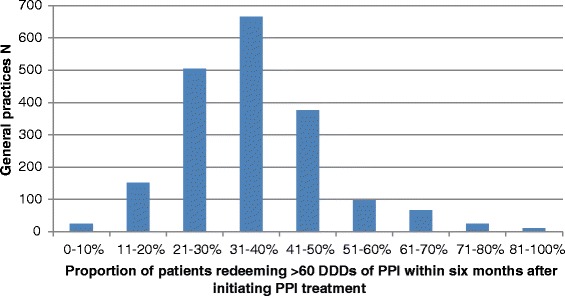
Distribution of the *long-term user proportion* among general practices in total numbers (*N* = 1919)

The distribution of the *“long-term user proportion”* among general practices is illustrated in Fig. 3 and demonstrates a large variation among practices in the proportion of patients redeeming more than 60 DDDs of PPI within six months after starting PPI treatment.

Table 1 illustrates the general practice characteristics and the corresponding mean *“long-term user proportion”*.

**Table 1:** Distribution of prescriber characteristics within the entire general practice cohort. The mean and standard deviation (SD) of the variable “long-term user proportion”* is reported for each prescriber characteristic

	N all practices	Mean (SD)	N single-handed	Mean (SD)	N partnership	Mean (SD)
Practice organisation						
Single-handed	1041	0.37 (0.15)	1041	0.37 (0.15)	0	-
Partnership	878	0.33 (0.09)	0	-	878	0.33 (0.09)
Training practice						
No	1286	0.36 (0.14)	823	0.37 (0.16)	463	0.33 (0.10)
Yes	633	0.35 (0.11)	218	0.36 (0.14)	415	0.34 (0.09)
Number of GPs						
1	1041	0.37 (0.15)	1041	0.37 (0.15)	0	-
2	414	0.33 (0.11)	0	-	414	0.33 (0.11)
3	246	0.33 (0.09)	0	-	246	0.33 (0.09)
4	129	0.33 (0.08)	0	-	129	0.33 (0.08)
5	56	0.33 (0.08)	0	-	56	0.33 (0.08)
>5	33	0.33 (0.06)	0	-	33	0.33 (0.06)
GP age group						
Under 45 years	121	0.32 (0.11)	48	0.33 (0.14)	73	0.31 (0.09)
45-49 years	261	0.32 (0.09)	68	0.33 (0.11)	193	0.31 (0.08)
50-54 years	455	0.33 (0.11)	150	0.33 (0.14)	305	0.32 (0.09)
55-59 years	497	0.36 (0.13)	285	0.36 (0.15)	212	0.34 (0.09)
60-64 years	385	0.39 (0.16)	313	0.39 (0.16)	72	0.38 (0.11)
65 years or above	200	0.40 (0.15)	177	0.40 (0.15)	23	0.40 (0.13)
GP gender						
Male	875	0.38 (0.15)	735	0.38 (0.15)	140	0.38 (0.11)
Predominantly male	193	0.34 (0.08)	0	-	193	0.34 (0.08)
Equally mixed	302	0.32 (0.09)	0	-	302	0.32 (0.09)
Predominantly female	153	0.32 (0.08)	0	-	153	0.32 (0.08)
Female	396	0.33 (0.14)	306	0.34 (0.15)	90	0.30 (0.09)
Practice location						
Capital area	602	0.34 (0.14)	412	0.36 (0.15)	190	0.32 (0.11)
Provincial city	760	0.35 (0.13)	388	0.37 (0.16)	372	0.32 (0.09)
Rural area	557	0.37 (0.12)	241	0.39 (0.15)	316	0.35 (0.09)
Patients per GP						
<1350	575	0.35 (0.13)	192	0.38 (0.16)	383	0.33 (0.10)
1350-1575	453	0.36 (0.14)	249	0.38 (0.17)	204	0.34 (0.09)
1576-1750	414	0.35 (0.13)	247	0.37 (0.16)	167	0.33 (0.08)
>1750	477	0.35 (0.13)	353	0.36 (0.13)	124	0.32 (0.10)

*The “long-term user proportion” is defined as the proportion of adult first time users of PPI within the practice who redeemed more than 60 DDDs of PPI within six months in 2011

When comparing all general practices, univariate regression analyses revealed that initiating long-term treatment with PPIs was significantly associated with GPs being male, 55 years and above, single-handed practice and practicing in rural locations (Table 2).

**Table 2:** Crude and adjusted odds ratios (ORs) with 95 % confidence intervals (CI) for the associations between characteristics of all general practices and initiating long-term treatment with PPIs

	Crude OR (95 % CI)	*P*-value	Adjusted OR* (95 % CI)	*P*-value
Patients per GP				
<1350	1	.	1	.
1350-1575	1.03 (0.96;1.10)	0.43	1.00 (0.93;1.07)	0.90
1576-1750	1.00 (0.94;1.08)	0.92	1.01 (0.94;1.09)	0.74
>1750	1.01 (0.94;1.08)	0.83	1.00 (0.93;1.07)	0.94
Geography				
Capital area	1	.	1	.
Provincial city	1.01 (0.95;1.07)	0.73	1.02 (0.96;1.09)	0.52
Rural area	1.11 (1.04;1.19)	<0.001	1.04 (0.97;1.11)	0.23
GP gender				
Male	1	.	1	.
Predominantly male	0.81 (0.75;0.87)	<0.001	0.88 (0.78;1.01)	0.07
Equally mixed	0.77 (0.72;0.82)	<0.001	0.83 (0.76;0.91)	<0.001
Predominantly female	0.76 (0.70;0.83)	<0.001	0.87 (0.76;1.00)	0.04
Female	0.77 (0.71;0.82)	<0.001	0.87 (0.81;0.93)	<0.001
GP age group				
Under 45 years	1	.	1	.
45-49 years	1.02 (0.91;1.13)	0.79	0.99 (0.88;1.11)	0.86
50-54 years	1.06 (0.95;1.17)	0.29	1.01 (0.91;1.12)	0.89
55-59 years	1.21 (1.09;1.34)	<0.001	1.12 (1.00;1.24)	0.04
60-64 years	1.44 (1.29;1.61)	<0.001	1.30 (1.16;1.46)	<0.001
65 years or above	1.46 (1.29;1.65)	<0.001	1.32 (1.16;1.50)	<0.001
Number of GPs				
1	1	.	1	.
2	0.86 (0.81;0.92)	<0.001	1.04 (0.95;1.13)	0.40
3	0.83 (0.78;0.89)	<0.001	1.00 (0.88;1.14)	0.99
4	0.83 (0.76;0.91)	<0.001	1.05 (0.92;1.19)	0.50
5	0.85 (0.75;0.96)	0.01	1.01 (0.84;1.20)	0.94
>5	0.84 (0.72;0.98)	0.03	1.01 (0.83;1.22)	0.94
Training practice				
No	1	.	1	.
Yes	0.98 (0.93;1.03)	0.36	1.04 (0.98;1.09)	0.20

*Estimates adjusted for all other practice characteristics and patient characteristics in terms of age, gender, specific gastrointestinal morbidity, socioeconomic status (income, educational level, cohabitation status), comedication with non-steroidal anti-inflammatory drugs (NSAIDs), antiplatelet drugs, anticoagulants, selective serotonine reuptake inhibitors (SSRIs) and comorbidity

Only GPs being male and aged 55 years and above were independently and significantly associated with initiating long-term treatment with PPIs in the adjusted analyses (Table 2). These associations remained statistically significant, when stratifying the analyses into single-handed and partnership practices, except for GP age group in single-handed practices where only age of 60 years and above was significantly associated with initiating long-term treatment with PPIs (Tables 3 and 4). Similar associations were found in the sensitivity analysis with the definition of long-term use increased to >90 DDDs within six months, i.e. increasing the threshold for long-term use changed neither the direction nor the magnitude of the associations substantially.

**Table 3:** Crude and adjusted odds ratios (OR) with 95 % confidence intervals (CI) for the association between characteristics of single-handed practices and initiating long-term treatment with PPIs

	Crude OR (95 % CI)	*P*-value	Adjusted OR* (95 % CI)	*P*-value
Patients per GP				
<1350	1	.	1	.
1350-1575	0.96 (0.84;1.11)	0.60	1.02 (0.88;1.17)	0.81
1576-1750	0.95 (0.83;1.09)	0.45	1.07 (0.92;1.23)	0.38
>1750	0.91 (0.80;1.03)	0.13	1.04 (0.91;1.20)	0.53
Practice location				
Capital area	1	.	1	.
Provincial city	1.08 (0.98;1.19)	0.13	1.06 (0.96;1.17)	0.29
Rural area	1.18 (1.06;1.31)	<0.001	1.05 (0.94;1.18)	0.37
GP gender				
Male	1	.	1	.
Female	0.79 (0.72;0.87)	<0.001	0.89 (0.81;0.99)	0.03
GP age group				
Under 45 years	1	.	1	.
45-49 years	1.02 (0.80;1.30)	0.88	0.95 (0.73;1.22)	0.67
50-54 years	1.02 (0.82;1.27)	0.83	0.96 (0.77;1.21)	0.74
55-59 years	1.19 (0.97;1.45)	0.10	1.11 (0.90;1.37)	0.35
60-64 years	1.39 (1.14;1.71)	<0.001	1.26 (1.02;1.56)	0.03
65 years or above	1.38 (1.12;1.71)	<0.001	1.29 (1.03;1.62)	0.03
Training practice				
No	1	.	1	.
Yes	0.96 (0.87;1.07)	0.46	0.96 (0.86;1.07)	0.45

*Estimates adjusted for practice characteristics and patient characteristics in terms of age, gender, specific gastrointestinal morbidity, socioeconomic status (income, educational level, cohabitation status), comedication with non-steroidal anti-inflammatory drugs (NSAIDs), antiplatelet drugs, anticoagulants, selective serotonine reuptake inhibitors (SSRIs) and comorbidity

**Table 4:** Crude and adjusted odds ratios (OR) with 95 % confidence intervals (CI) for the association between characteristics of partnership practices and initiating long-term treatment with PPIs

	Crude OR (95 % CI)	*P*-value	Adjusted OR* (95 % CI)	*P*-value
Patients per GP				
<1350	1	.	1	.
1350-1575	1.00 (0.93;1.07)	0.94	0.99 (0.93;1.06)	0.83
1576-1750	0.95 (0.88;1.03)	0.19	0.98 (0.91;1.06)	0.69
>1750	0.95 (0.88;1.04)	0.27	0.98 (0.90;1.06)	0.61
Practice location				
Capital area	1	.	1	.
Provincial city	1.01 (0.94;1.09)	0.75	0.98 (0.90;1.05)	0.53
Rural area	1.15 (1.06;1.24)	<0.001	1.01 (0.94;1.10)	0.74
GP gender				
Male	1	.	1	.
Predominantly male	0.82 (0.75;0.89)	<0.001	0.85 (0.76;0.95)	<0.001
Equally mixed	0.78 (0.71;0.85)	<0.001	0.80 (0.73;0.87)	<0.001
Predominantly female	0.77 (0.70;0.85)	<0.001	0.83 (0.74;0.94)	<0.001
Female	0.70 (0.62;0.79)	<0.001	0.78 (0.69;0.88)	<0.001
GP age group				
Under 45 years	1	.	1	.
45-49 years	1.03 (0.92;1.15)	0.58	1.02 (0.91;1.14)	0.75
50-54 years	1.08 (0.97;1.21)	0.14	1.04 (0.93;1.15)	0.50
55-59 years	1.21 (1.08;1.35)	<0.001	1.13 (1.01;1.26)	0.03
60-64 years	1.42 (1.23;1.63)	<0.001	1.34 (1.16;1.54)	<0.001
65 years or above	1.54 (1.26;1.87)	<0.001	1.35 (1.11;1.64)	<0.001
Number of GPs				
2	1	.	1	.
3	0.96 (0.90;1.03)	0.26	0.95 (0.86;1.06)	0.37
4	0.96 (0.89;1.04)	0.36	0.99 (0.91;1.09)	0.91
5	0.98 (0.88;1.09)	0.71	0.96 (0.83;1.10)	0.52
>5	0.96 (0.85;1.10)	0.58	0.95 (0.82;1.10)	0.48
Training practice				
No	1	.	1	.
Yes	1.07 (1.01;1.13)	0.03	1.08 (1.02;1.14)	<0.001

*Estimates adjusted for practice characteristics and patient characteristics in terms of age, gender, specific gastrointestinal morbidity, socioeconomic status (income, educational level, cohabitation status), comedication with non-steroidal anti-inflammatory drugs (NSAIDs), antiplatelet drugs, anticoagulants, selective serotonine reuptake inhibitors (SSRIs) and comorbidity

In the stratified analyses, a positive association between training practice status and initiating long-term treatment with PPIs was statistically significant for partnership practices (OR 1.08, CI 1.02-1.14).

## Discussion

### Main findings

Male gender and increasing age of GPs are significantly associated with initiating long-term treatment with PPIs in a cohort of first-time users treated in a primary care setting. However, general practice characteristics do not seem to be strong predictors of initiating long-term treatment with PPIs as the magnitude of the associations is modest. The apparent variation with regard to rurality and partnership status observed in the crude analyses was no longer statistically significant when adjusting for practice- and patient-related factors. Given that the patient population in rural and single-handed practices is older and has a higher degree of morbidity [29, 30], this might reflect a different composition of patient populations in different practice types and locations.

### Strengths and limitations

The major strength of this study is the register-based design enabling us to include information on all GPs issuing first-time prescriptions of PPI to a nationwide cohort of incident PPI users. The validity of the register data is considered high [20], and the accurate linkage between the patient-related factors and the practice characteristics makes it possible to adjust for numerous predisposing patient factors and thereby isolate and analyse the independent associations between practice characteristics and initiation of long-term treatment with PPIs. Only 3 % of the total sale of PPI in Denmark is over-the-counter [1], and therefore registration of PPI use is considered almost complete.

The fact that we extracted practice data at the time of initiating long-term treatment with PPIs provides the study with the strength to accurately identify factors of the physician responsible for initiation of long-term prescribing and to characterise practice features as predictors for incident long-term prescribing. In case we had analysed practice characteristics associated with prevalent long-term prescribing of PPIs, we would not have been able to be sure of the validity of the predictors, because prevalent long-term prescribing might have been established several years earlier by another physician.

However, influence of unmeasured factors cannot be ruled out. The linkage between the prescription and the physician factors can only be made at practice level, impeding identification of the GP within partnership practices primarily providing care to the patient. This limitation makes it difficult to assess the effects of doctors’ age and gender in partnership practices. Exact information on age and gender would have been more precise than the mean age of doctors within the practice and the relative gender distribution. Therefore, the associations between long-term prescribing of PPIs and GP age and gender can only be certain for single-handed practices, though the associations seem similar for partnership practices.

Other potential influential variables could have been interesting to include in the analyses had they been available. Practice characteristics such as distance to outpatient clinics (i.e. endoscopy, gastroenterological expertise etc.) could influence prescribing of PPIs. However, data on practice location are limited to postcodes in order to keep the individual prescriber unidentifiable, and the exact address is not available to calculate distance to outpatient clinics.

### Interpretation of findings in relation to previously published studies

To the best of our knowledge no other study has investigated the associations between practice characteristics and prescribing of PPIs. Our study does not clarify why older GPs to a greater extent prescribe long-term treatment with PPIs in first-time users, but it is important to keep in mind that few single-handed practices are run by younger GPs (Table 1). These practices could have a smaller *long-term user proportion* due to random variation, decreasing the comparability between younger and older GPs in single-handed practices. Nevertheless, in line with our findings it has been demonstrated that older GPs have higher prescribing rates [14, 31, 32] and lower rates of non-pharmacological treatments [14]. Similar to our analyses, other studies have found that GPs of male gender have higher prescribing rates [31, 33]. This could be due to the traditional thought of female GPs being more psychosocially orientated and more patient-centred compared to male GPs [34, 35].

In the multivariate analyses we found no association between single-handed practices and high rate of initiating long-term prescribing of PPIs. In some studies partnership practices have been associated with higher scores for quality of care in chronic diseases [12, 36], although the opposite has been shown as well [37, 38]. Moreover, patient satisfaction seems to be higher in single-handed practices [39]. However, in the present study we cannot determine either quality of care or patient satisfaction.

Being a training practice has also been shown to influence management of patients and quality of care [12, 40]. In our study we saw no association between training practice status and initiating long-term treatment with PPIs except for partnership practices, where training practices had slightly higher odds of initiating long-term treatment (OR 1.08, CI 1.02-1.14). Why training practice status influences partnership practices, but not single-handed practices, is unknown. The association found is, however, fairly weak and just barely statistically significant.

Rurality has been associated with higher rates of inappropriate prescribing [41] and different prescribing patterns [42]. That we do not find similar associations could be due to the fact that Denmark is a quite homogenous country with small distances and less difference between rural and urban areas compared to other countries.

## Conclusion

Overall we conclude that there was significant variation in initiating long-term treatment with PPIs between practices. Some of this variation was associated with GP characteristics despite part of the variation being due to differences in characteristics of patient population. This underlines the importance of taking patient characteristics into account, when assessing associations between GP characteristics and prescribing rates.

Whether or not initiating prescribing of long-term treatment with PPIs reflects quality in the clinical setting is challenging to determine in this study, but our results indicate that older GPs and GPs of male gender might have lower threshold for initiating long-term treatment with PPIs, though the associations found were modest. Some of the variation could be due to differences in thoughts and knowledge about PPIs [43, 44]. The demonstration of a variation does not in itself require interventions, but the findings could contribute to evidence-based design of future interventions to enhance rational prescribing of PPIs. The results call for reflection and awareness of the fact that the care delivered is influenced by the health care provider. To fully understand the variations in initiating long-term use of PPIs, future research could benefit from moving beyond the aggregate associations and further explore the mental and social processes at work.

## Abbreviations

CI, Confidence interval; DDDs, Defined daily doses; GP, General Practitioner; NSAIDs, Non-steroid anti-inflammatory drugs; OR, Odds ratio; PPIs, Proton pump inhibitors; SSRIs, Selective serotonin reuptake inhibitors.
